# Respiratory Syncytial Virus Immunization Coverage Among Infants Through Receipt of Nirsevimab Monoclonal Antibody or Maternal Vaccination — United States, October 2023–March 2024

**DOI:** 10.15585/mmwr.mm7431a3

**Published:** 2025-08-21

**Authors:** Ellen O. Boundy, Hannah Fast, Tara C. Jatlaoui, Hilda Razzaghi, LaTreace Harris, Kimberly Nguyen, Jamie Mells, Georgina Peacock, Carla L. Black

**Affiliations:** ^1^Immunization Services Division, National Center for Immunization and Respiratory Diseases, CDC; ^2^U.S. Public Health Service Commissioned Corps, Rockville, Maryland.

**Summary**

**What is already known about this topic?**

Respiratory syncytial virus (RSV) is the most common cause of hospitalization among U.S. infants. In 2023, a long-acting monoclonal antibody for infants (nirsevimab) and a maternal vaccine were recommended to prevent RSV among infants. In the same year, data from state- and jurisdiction-level immunization information systems (IISs) became available at CDC.

**What is added by this report?**

Cross-sectional analysis of data from IISs representing 33 states and the District of Columbia found that 29% of infants born during October 2023–March 2024 were immunized against RSV through receipt of nirsevimab or through maternal RSV vaccination during pregnancy in the 2023–24 respiratory virus season, the first season that these products were available. State-specific immunization coverage from nirsevimab or maternal vaccination ranged from 11% to 53%.

**What are the implications for public health practice?**

Continued efforts are needed to increase infant RSV immunization coverage and reduce associated morbidity. IISs are population-based data sources that can be used to monitor jurisdiction-level coverage.

## Abstract

Respiratory syncytial virus (RSV) is the leading cause of infant hospitalization in the United States. A new RSV monoclonal antibody (nirsevimab) for infants and an RSV vaccine for pregnant women were recommended by the Advisory Committee on Immunization Practices in August and September 2023, respectively, to protect infants against RSV infection. Sufficient data have become available to allow estimates of infant RSV immunization coverage through administration of these products. Among infants born during October 2023–March 2024, infant RSV immunization coverage was estimated by summing the total number of infants who received nirsevimab and the number of women of childbearing age who received RSV vaccine, as reported to immunization information systems (IISs) in 33 U.S. states and the District of Columbia (DC), and dividing by the total number of live births, obtained from CDC Wide-ranging Online Data for Epidemiologic Research (WONDER) natality data. Across 33 states and DC, an estimated 29% of infants born during October 2023–March 2024 were immunized against RSV during the 2023–24 respiratory virus season, including 19% through infant receipt of nirsevimab and 10% through maternal RSV vaccination. Infant RSV immunization coverage through nirsevimab or maternal vaccination ranged from an estimated 11% to 53% by state. Among infants who received nirsevimab, 38% received it within the first week of life (0–6 days after birth). Continued efforts are needed to increase RSV immunization coverage among infants and pregnant women.

## Introduction

Respiratory syncytial virus (RSV) is the leading cause of hospitalization among U.S. infants (*1*). On August 3, 2023, CDC’s Advisory Committee on Immunization Practices (ACIP) recommended use of nirsevimab, a long-acting monoclonal antibody, for all infants aged <8 months born during or entering their first RSV season (October–March* for most of the continental United States) (*2*). On September 22, 2023, ACIP recommended administration of 1 dose of RSV vaccine (Abrysvo, Pfizer Inc.) to pregnant women at 32–36 gestational weeks during September–January (for most of the continental United States) to provide protection to infants aged <6 months through transplacental transfer of maternal antibodies (*3*). Only one of these products, either nirsevimab for the infant or vaccination for the mother, is recommended for each pregnant woman and her infant, except in rare instances (*3*). Since the approval of nirsevimab for infants and the maternal RSV vaccine, sufficient data have become available to allow estimates of infant RSV immunization coverage through administration of these products.

Immunization information systems (IISs) are confidential, population-based systems that collect immunization administration data from health care providers in a given U.S. jurisdiction.^†^ Individual jurisdictions began submitting IIS data for all routine immunizations to CDC quarterly in 2023. These data include recipient demographic characteristics and administration information for each immunization provided, including information on child eligibility for the Vaccines for Children (VFC) program,^§^ which provides vaccines at no cost to uninsured and underinsured children through enrolled health care providers and includes coverage for nirsevimab.^¶^ In this report, data were analyzed to estimate the proportion of infants born during October 2023–March 2024 who received RSV immunization either through infant nirsevimab administration or maternal RSV vaccination during the 2023–24 respiratory virus season, among U.S. jurisdictions reporting IIS data to CDC. Several studies have reported on nirsevimab and maternal RSV vaccine use in the first season after their approval in a single U.S. hospital or state (*4*–*7*). This report is the first CDC analysis of RSV immunization coverage using a population-based data source for numerous states.

## Methods

### Data Source

IIS data were analyzed to assess 1) administration of nirsevimab during October 1, 2023–March 31, 2024, to infants born during this period and 2) administration of RSV vaccine (Abrysvo) during September 1, 2023–January 31, 2024, to women aged 18–49 years, according to recommended timelines (*2*,*3*). Women aged 18–49 years who received RSV vaccination were presumed to be pregnant because RSV vaccine is only recommended for women in this age group who are pregnant.

The total number of live births was obtained from CDC Wide-ranging Online Data for Epidemiologic Research (WONDER) natality data.** Data were collected from 36 jurisdictions (33 states, two localities [New York City and Philadelphia], and the District of Columbia [DC]) that submitted deidentified, line-level IIS data to CDC for immunizations received through March 31, 2024. IIS data from New York City and Philadelphia were combined with their state data from New York and Pennsylvania, respectively, for an analytic sample of 33 states and DC. Among these jurisdictions, two (Montana and Pennsylvania) have an opt-in consent policy both for children and adults that requires explicit consent to be provided for their data to be included in the IIS. Two other jurisdictions (New York and New York City) have opt-in consent policies only for adults aged ≥19 years. All other included jurisdictions have opt-out or mandatory inclusion policies.

### Analysis

The overall and state-specific percentages of infants covered by one of the two RSV products were estimated by summing the number of infants who received at least 1 dose of nirsevimab and the total number of women aged 18–49 years who received at least 1 dose of RSV vaccine (as reported to the jurisdictional IIS) and dividing the total by the number of live births to women in this age group during October 2023–March 2024 (from CDC WONDER natality data), calculated by state. Percentages were also stratified by recipient characteristics, including age, month of immunization receipt, VFC program eligibility, and infant birth month. SAS software (version 9.4; SAS Institute) and Azure Databricks (Version 14.0, Databricks Runtime) were used to conduct all analyses. This activity was reviewed by CDC, deemed not research, and was conducted consistent with applicable federal law and CDC policy.^††^

## Results

### Administration of Nirsevimab and Maternal RSV Vaccine

During the 2023–24 respiratory virus season, across 33 states and DC, 213,659 infants born during October–March received nirsevimab, and 119,879 women aged 18–49 years received RSV vaccine (Table) (Supplementary Table). The largest percentages of nirsevimab doses were administered to infants in December (20.6%), January (20.2%), and February (20.2%), and the largest percentages of maternal RSV vaccine doses were administered in December (31.6%) and January (36.2%).

**TABLE:** Number and percentage of infants immunized against respiratory syncytial virus through receipt of nirsevimab monoclonal antibody or maternal vaccination, by demographic characteristics — 33 states and District of Columbia,* October 2023–March 2024

Characteristic	No. (%)	
	Infants who received nirsevimab^†^	Pregnant women who received RSV vaccine^§^
**Total, no.**	**213,659**	**119,879**
**Infant’s age at nirsevimab receipt**		
0–3 days	44,510 (20.8)	—
4–6 days	36,818 (17.2)	—
7 days–<1 mo	65,009 (30.4)	—
1 mo	33,160 (15.5)	—
2–5 mos	34,162 (16.0)	—
**Mother’s age at RSV vaccine receipt, yrs**		
18–24	—	13,105 (10.9)
25–34	—	70,921 (59.2)
35–49	—	35,853 (29.9)
**Immunization month and year **		
Sept 2023	—	501 (0.4)
Oct 2023	9,096 (4.3)	10,235 (8.5)
Nov 2023	39,324 (18.4)	27,866 (23.2)
Dec 2023	44,095 (20.6)	37,938 (31.6)
Jan 2024	43,245 (20.2)	43,339 (36.2)
Feb 2024	43,142 (20.2)	—
Mar 2024	34,757 (16.3)	—
**Vaccines for Children program eligibility** **^¶^**		
Eligible	91,817 (43.0)	—
Ineligible	74,907 (35.1)	—
Unknown	46,935 (22.0)	—
**Infant’s birth month and year**		
Oct 2023	47,020 (22.0)	—
Nov 2023	46,507 (21.8)	—
Dec 2023	40,941 (19.2)	—
Jan 2024	34,008 (15.9)	—
Feb 2024	27,377 (12.8)	—
Mar 2024	17,806 (8.3)	—

**Abbreviation:** RSV = respiratory syncytial virus.

* Includes Alaska, Arizona, Arkansas, California, Connecticut, Delaware, Florida, Illinois, Indiana, Iowa, Kentucky, Louisiana, Maine, Maryland, Michigan, Minnesota, Mississippi, Missouri, Montana, Nevada, New Mexico, New York, Ohio, Oklahoma, Pennsylvania, South Dakota, Tennessee, Utah, Vermont, Washington, West Virginia, Wisconsin, and Wyoming.

^†^ Includes infants born during October 1, 2023–March 31, 2024, who received at least 1 dose of nirsevimab during October 1, 2023–March 31, 2024.

^§^ Includes women (defined in the Immunization Information Systems as persons whose sex was female) who were aged 18–49 years at the time of RSV vaccination and received the vaccine (Abrysvo) during September 1, 2023–January 31, 2024. These women were presumed to be pregnant because RSV vaccination is only recommended for women in this age group if they are pregnant.

^¶^ The Vaccines for Children program provides vaccines at no cost to uninsured and underinsured children through enrolled public and private health care providers.

### Overall and State-Specific Percentages of Infants Immunized Through Nirsevimab or Maternal RSV Vaccination

**FIGURE 1:**
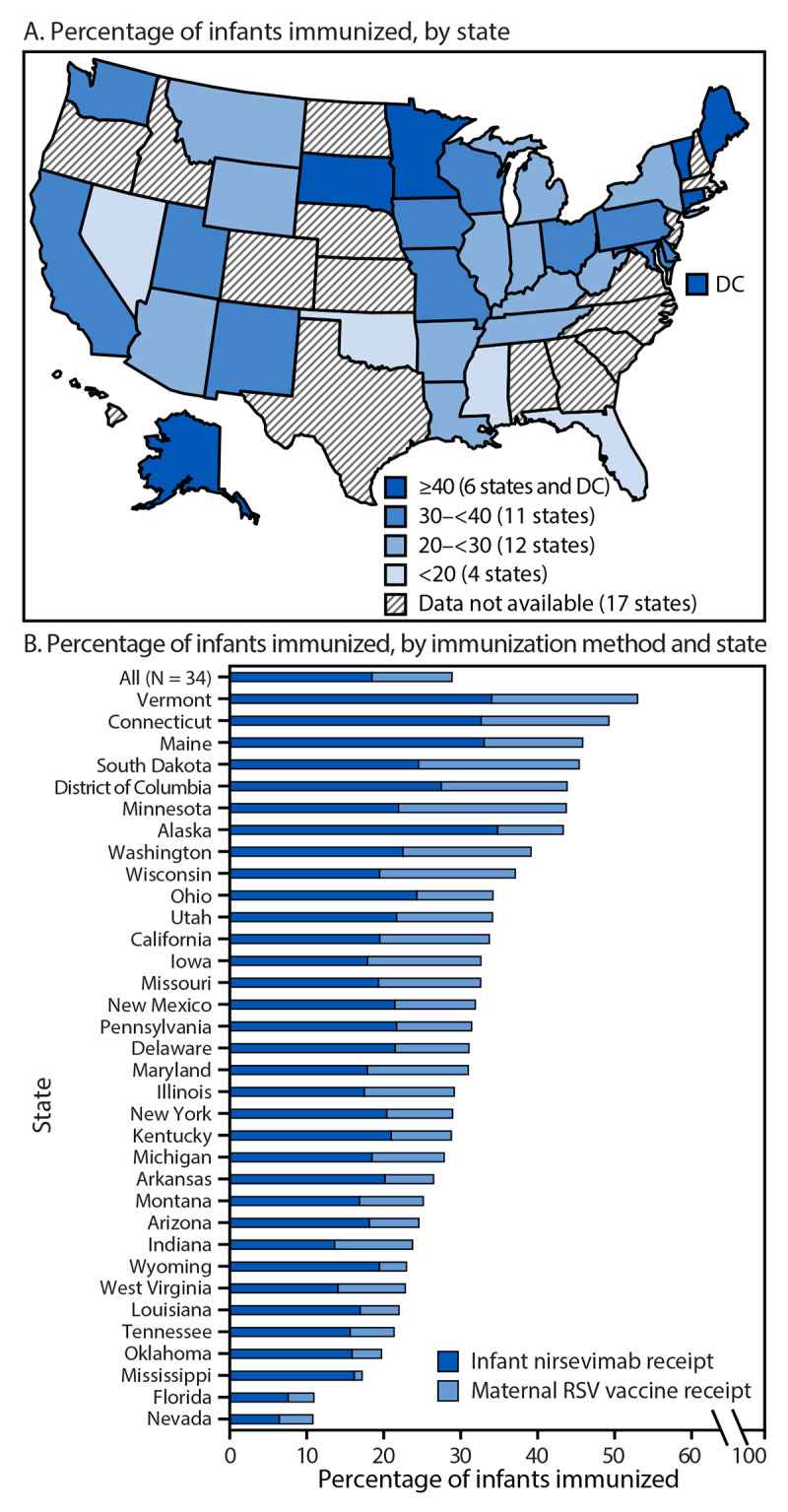
Percentage of infants* immunized against respiratory syncytial virus through receipt of nirsevimab^†^ or maternal vaccination,^§^ by state (A) and method of immunization (B) — 33 states and District of Columbia, October 2023–March 2024 **Abbreviations:** DC = District of Columbia; RSV = respiratory syncytial virus. * The denominator includes infants born during October 2023–March 2024 to mothers aged 18–49 years. Although infants born to younger and older mothers were not included for calculations of infant protection from RSV, nirsevimab doses received by these infants could not be identified and removed from the numerator. ^†^ Calculated as the number of infants who received at least 1 dose of nirsevimab during October 1, 2023–March 31, 2024, divided by the number of infants born during October 2023–March 2024 to mothers aged 18-49 years. ^§^ Calculated as the number of women aged 18–49 years who received at least 1 dose of RSV vaccine (Abrysvo) during September 1, 2023–January 31, 2024, divided by the number of infants born during October 2023–March 2024 to mothers aged 18–49 years.

Across 33 states and DC, 28.9% of infants born during October 2023–March 2024 were immunized through either nirsevimab (18.5%) or maternal RSV vaccination (10.4%) (Figure 1) (Supplementary Table). By state, coverage ranged from 10.8% in Nevada to 53.1% in Vermont. Infant nirsevimab coverage ranged from 6.5% in Nevada to 34.9% in Alaska, and maternal RSV vaccination coverage ranged from 1.0% in Mississippi to 21.8% in Minnesota. Six states (Alaska, Connecticut, Maine, Minnesota, South Dakota, and Vermont) and DC reported that approximately one half of infants were immunized through either nirsevimab or maternal vaccination (range = 43.4%–53.1%), and four states (Florida, Mississippi, Nevada, and Oklahoma) reported that fewer than one fifth of infants were immunized (range = 10.8%–19.7%).

### Nirsevimab Coverage During the Respiratory Virus Season 

**FIGURE 2:**
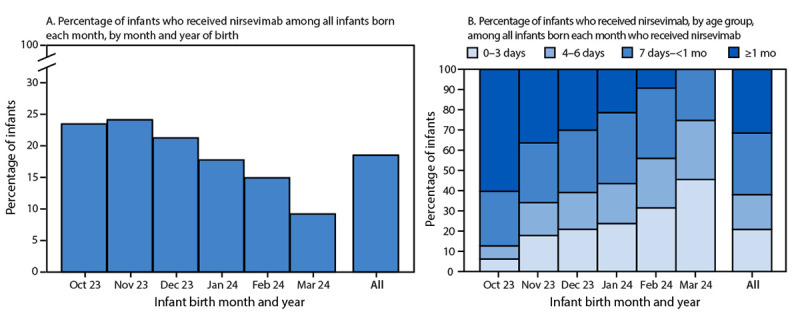
Percentage of infants immunized against respiratory syncytial virus through receipt of nirsevimab,* by month and year of birth (A) and age group (B) — 33 states and District of Columbia,^†^ October 2023–March 2024 * Calculated as the number of infants who received at least 1 dose of nirsevimab during October 1, 2023–March 31, 2024, divided by the number of infants born each month during October 2023–March 2024 to mothers aged 18–49 years. ^†^ Includes Alaska, Arizona, Arkansas, California, Connecticut, Delaware, Florida, Illinois, Indiana, Iowa, Kentucky, Louisiana, Maine, Maryland, Michigan, Minnesota, Mississippi, Missouri, Montana, Nevada, New Mexico, New York, Ohio, Oklahoma, Pennsylvania, South Dakota, Tennessee, Utah, Vermont, Washington, West Virginia, Wisconsin, and Wyoming.

By infant birth month, nirsevimab coverage was 23.8% among infants born during October–November 2023 and decreased toward the end of the respiratory virus season to 9.2% among infants born in March 2024 (Figure 2). Among all infants who received nirsevimab, 38.1% of doses were administered within the first 6 days of life, 30.4% within 7 days–<1 month, and 31.5% at age ≥1 month. Infants born toward the end of the respiratory virus season who received nirsevimab were more likely to receive the antibodies within the first 3 days of life (45.5% of infants born in March) than were those born at the start of the season (6.2% of infants born in October).

### Nirsevimab Coverage and VFC Program Eligibility

Among infants who received nirsevimab, 43.0% were eligible for the VFC program, 35.1% were not eligible, and 22.0% did not have VFC information available in IIS (Table). A lower percentage of infants who received nirsevimab within the first 3 days of life were eligible for the program (29.9%) than were those who received nirsevimab at age ≥1 month (50.3%) (Supplementary Figure).

## Discussion

This report is the first analysis conducted at CDC to provide population-based estimates of the percentage of infants who were immunized against RSV in numerous U.S. states and to analyze the timing of immunization. IIS data from 33 states and DC found that during the first season after approval of nirsevimab for infants and an RSV vaccine for pregnant women, 28.9% of infants born during October 2023–March 2024 were immunized against RSV, either through receipt of nirsevimab or through maternal RSV vaccination. The percentages of infants covered varied widely by state, from 10.8% to 53.1%. Only 38.1% of infants who received nirsevimab received the antibodies within the first week of life (0–6 days after birth), the optimal timing for maximum protection.^§§^

An analysis of data from an internet panel survey conducted by CDC during March 26–April 11, 2024, estimated that approximately double the percentage (55.8%) of U.S. infants received protection against RSV through maternal RSV vaccination and nirsevimab during the 2023–24 season as the percentage estimated in this report (*8*). Differences between the findings of that study and those in this report might be due to an overestimation of coverage in the survey (from self-selection bias, recall bias, or limited sample size) or an underestimation in IIS-based estimates of infant protection in some states because of jurisdictional policies and variation in reporting by immunization providers that might limit the capture of all immunizations administered.^¶¶^ Some RSV immunization providers, including birthing hospitals (those with more than one birth within the previous year or at least one registered maternity bed) and outpatient obstetric health care providers who administer immunizations less frequently, might not participate in their jurisdictional IIS, which might limit the data that were used in this report. Several other recent studies (*4*–*6*,*9*) also reported higher immunization coverage estimates than those in this report; however, most of those analyses were from a single institution or health care network with targeted interventions to promote RSV immunization. A population-based study in Wisconsin using IIS data reported coverage estimates similar to those in this report (36.2%) (*7*).

Several factors might have contributed to low RSV immunization coverage during the 2023–24 respiratory virus season. First, although nirsevimab was recommended in August 2023, supply issues limited its availability in some areas, particularly at the beginning of the season (*10*). Second, lack of familiarity among patients and providers about nirsevimab for infants and maternal RSV vaccine, as well as the complexity of the related pediatric and maternal recommendations, might have contributed to limited acceptance or delayed administration. Third, cost concerns might have also played a role. Private health insurers are allowed a 1-year grace period before they are required to cover ACIP-recommended vaccines under the Affordable Care Act.*** In addition, because nirsevimab might have been too expensive for some hospitals to include as part of routine newborn care, they might have opted not to stock it. Finally, this report only included immunizations administered during the ACIP-recommended months for most geographic areas (*2*,*3*); some doses might have been administered outside those months. Preliminary data from the 2024–25 respiratory virus season suggest RSV immunization coverage among infants aged <8 months through maternal vaccination or nirsevimab increased to 57% nationally.^†††^ Receipt of nirsevimab among infants during the first week of life was more common toward the end of the season, potentially indicating improvements in supply, administration in birthing hospitals, and increased familiarity with nirsevimab use, among other factors. Continued monitoring of when infants receive nirsevimab will be important during the upcoming respiratory virus season. Nirsevimab coverage by birth month decreased across the season, which might reflect shorter duration of eligibility to receive nirsevimab or increased opportunities for maternal vaccination among infants born later in the season.

The findings in this report indicate wide variation in both nirsevimab and maternal RSV vaccine use across U.S. states and DC. Differences in age at receipt of nirsevimab were also found according to infants’ VFC eligibility status, with VFC-eligible infants less likely to receive nirsevimab within 3 days of birth, the period in which most infants are likely to be in a hospital after their birth. This might be an indication of a limited number of birthing hospitals being accredited as VFC providers. Despite ACIP approval occurring shortly before the respiratory virus season and challenges around nirsevimab supply and insurance reimbursement, in six states and DC in this analysis, ≥40% of infants were covered by one of the RSV immunization products. Another monoclonal antibody (clesrovimab) was also recently approved for use by the Food and Drug Administration and recommended by ACIP for infants aged <8 months who are not immunized through maternal vaccination, allowing additional options to provide infant immunization against RSV in upcoming seasons.^§§§^ This baseline information from the first season after RSV immunization product approval can be used by pediatric and obstetric health care providers and public health professionals to guide focused immunization strategies for future respiratory virus seasons.

### Limitations

The findings in this report are subject to at least seven limitations. First, although adult women who received RSV vaccine (Abrysvo) were assumed to be pregnant, IIS data do not include pregnancy status. Therefore, inclusion of vaccinated women who were not pregnant would have resulted in an overestimation of coverage. Second, because maternal and infant records could not be linked through the deidentified IIS data reported to CDC, determining whether some infants received immunization coverage both through nirsevimab and maternal vaccination was not possible. The proportion of infants receiving immunization coverage was estimated by summing the total number of adult women who received RSV vaccine and the number of infants who received nirsevimab then dividing the total by the total number of live births. This might have led to overestimation of the numerator for infant coverage if a mother and infant collectively were immunized through both methods; however, this is only recommended in rare instances and has not been reported frequently in other studies (*5*,*7*,*9*). Third, IIS data do not identify multiple pregnancies (which account for approximately 3% of live births^¶¶¶^) or stillbirths (approximately 0.6% of births****). Thus, IIS dose data might not fully align with the denominator of live births by state. Fourth, although infants born to mothers aged <18 and >49 years were not included in the denominator for calculations of infant coverage, nirsevimab doses received by these infants could not be identified and removed from the numerator. This might have resulted in an overestimate of the percentage of infants covered; however, this is likely to represent a small number because births to adolescents and women aged ≥50 years represent a small proportion of total live births. Fifth, each jurisdiction’s IIS data might not include all doses administered because of consent and provider reporting policies, particularly among adult populations. This might have led to an underestimation of numerators for estimates of infant coverage. Sixth, 22% of infant records were missing VFC eligibility status, limiting the ability to fully interpret those data. Finally, because these results are from 33 states and DC, they might not be generalizable to the entire U.S. population.

### Implications for Public Health Practice

Additional efforts are needed to increase infant protection against severe RSV through maternal or infant immunization. Continued work is also needed to increase birthing hospital enrollment in VFC and improve obstetric provider reporting of immunizations to the IIS. IISs provide timely, population-based data that can be used to estimate state-level infant RSV immunization coverage and monitor trends. Preliminary data from the 2024–25 season suggest increases in RSV immunization coverage, and the recent ACIP recommendation for an additional monoclonal antibody, clesrovimab, could increase access to RSV protection for infants in the 2025–26 respiratory virus season.^††††^

## Footnotes

* Jurisdictions with RSV seasonality that differs from most of the continental United States are recommended to follow state, territorial, or local guidance on vaccination timing.

†
Immunization Information Systems | CDC

§
About the Vaccines for Children Program | CDC

¶
CDC’s Vaccines for Children Program Addendum: Special Considerations for Nirsevimab | CDC

**
CDC WONDER | Natality Information: Live Births | CDC

†† 45 C.F.R. part 46.102(l)(2), 21 C.F.R. part 56; 42 U.S.C. Sect. 241(d); 5 U.S.C. Sect. 552a; 44 U.S.C. Sect. 3501 et seq.

§§
RSV Immunization Guidance for Infants and Young Children | CDC

¶¶ Immunization Information Systems Policy and Legislation | CDC

***
How to Pay for Vaccines | Adult Vaccines | CDC

††† The estimates in the following reference for the 2024–25 season include infants born April 2024–March 2025; therefore, the period is not strictly comparable to that in the current report, which includes infants born October 2023–March 2024. Implementation and Uptake of Nirsevimab and Maternal Vaccine for Infant Protection from RSV | CDC

§§§ ACIP Recommendations | ACIP | CDC

¶¶¶ Multiple Births | National Center for Health Statistics | CDC

****
Data and Statistics on Stillbirth | Stillbirth | CDC

††††
Child Immunization Schedule Addendum | Vaccines & Immunizations | CDC
